# Crystal structure of a binuclear nickel(II) complex constructed of 1*H*-imidazo[4,5-*f*][1,10]phenanthroline and doubly deprotonated benzene-1,3,5-tri­carb­oxy­lic acid

**DOI:** 10.1107/S205698901500420X

**Published:** 2015-03-21

**Authors:** Ying Lv, Xiang-Rong Hao

**Affiliations:** a College of Chemistry, Tonghua Normal University, Tonghua, Jilin 134002, People’s Republic of China

**Keywords:** crystal structure, nickel(II) complex, binuclear cluster, 1*H*-imidazo[4,5-*f*][1,10]phenanthroline, benzene-1,3,5-tri­carboxyl­ic acid, hydrogen bonding, π–π stacking

## Abstract

The title complex, [Ni_2_(C_9_H_4_O_6_)_2_(C_13_H_8_N_4_)_2_(H_2_O)_4_]·2H_2_O, bis­(μ-5-carb­oxy­benzene-1,3-di­carboxyl­ato-κ^2^ *O* ^1^:*O* ^1′^)bis­[di­aqua(1*H*-imidazo[4,5-*f*][1,10]phenanthroline-κ^2^ *N* ^7^,*N* ^8^)nickel(II)] di­hydrate, was obtained under solvothermal conditions by the reaction of benzene-1,3,5-tricarboxylic acid (H_3_BTC) with Ni(NO_3_)_2_ in the presence of 1*H*-imidazo[4,5-*f*][1,10]phenanthroline (IP). The crystal has triclinic (*P*-1) symmetry with a centrosymmetric binuclear nickel(II) cluster. The Ni^II^ atom is coordinated by two N atoms from a chelating 1*H*-imidazo[4,5-*f*][1,10]phenanthroline ligand, two carboxyl­ate O atoms from two 5-carb­oxy­benzene-1,3-di­carboxyl­ate ligands and two water mol­ecules in a slightly distorted octa­hedral geometry. Two carboxyl­ate groups bridge two Ni^II^ cations, forming the binuclear complex. Extensive N—H⋯O, O—H⋯O and O—H⋯N hydrogen bonding is present in the crystal structure, forming a three-dimensional supermolecular framework. Weak π–π stacking is observed between parallel HBTC^2−^ and IP ring systems, the face-to-face separation being 3.695 (2) Å.

## Related literature   

For general background, see: Stephenson *et al.* (2008 ▸). For details of the synthesis, see: Liu *et al.* (2009 ▸); Wu *et al.* (1997 ▸); Yang *et al.* (2010 ▸); Che *et al.* (2013 ▸).
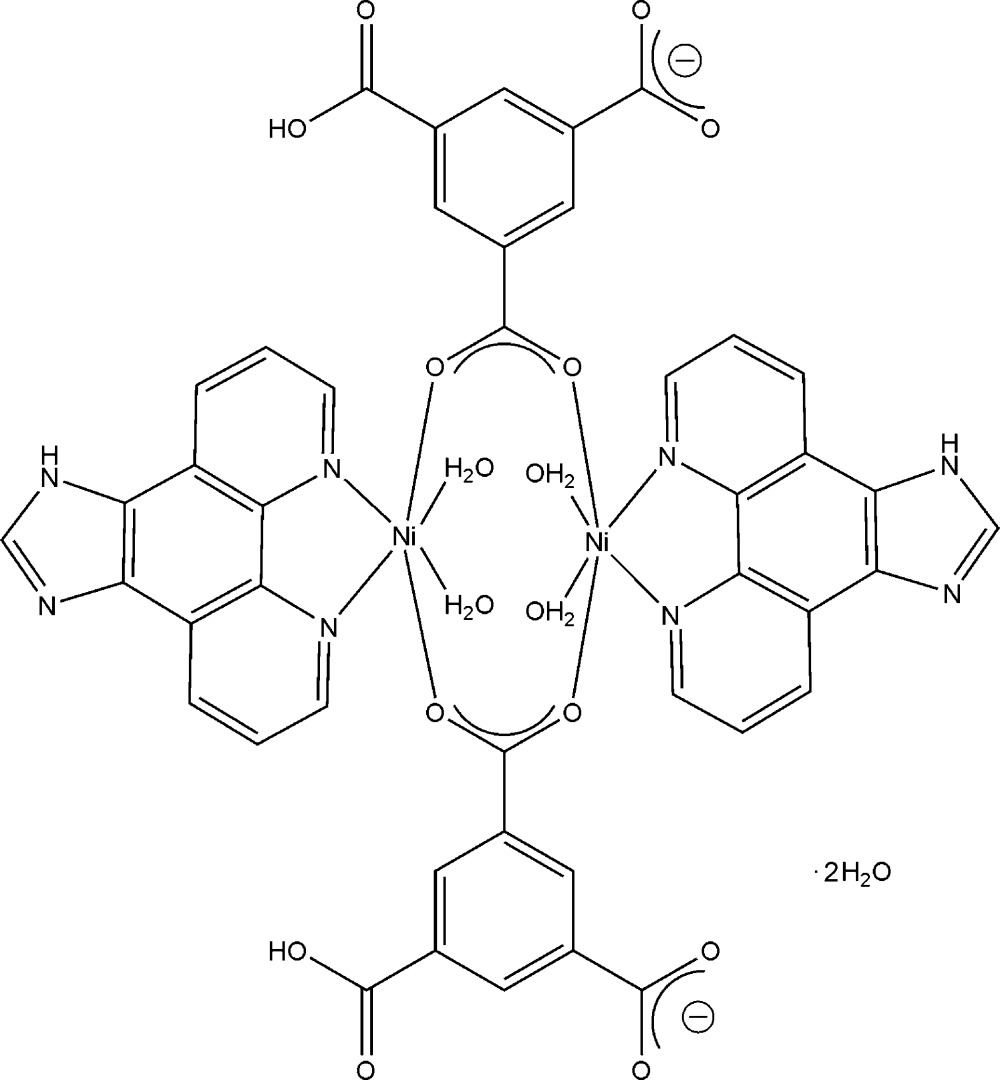


## Experimental   

### Crystal data  

[Ni_2_(C_9_H_4_O_6_)_2_(C_13_H_8_N_4_)_2_(H_2_O)_4_]·2H_2_O
*M* *_r_* = 1082.22
Triclinic, 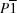

*a* = 8.581 (5) Å
*b* = 9.032 (5) Å
*c* = 14.278 (5) Å
α = 82.222 (5)°
β = 87.729 (5)°
γ = 73.117 (5)°
*V* = 1049.2 (9) Å^3^
*Z* = 1
Mo *K*α radiation
μ = 0.99 mm^−1^
*T* = 293 K
0.28 × 0.16 × 0.15 mm

### Data collection  

Bruker APEXII CCD diffractometer
Absorption correction: multi-scan (*SADABS*; Bruker, 2004 ▸) *T*_min_ = 0.805, *T*_max_ = 0.867
5594 measured reflections
3851 independent reflections
3050 reflections with *I* > 2σ(*I*)
*R*_int_ = 0.048

### Refinement  

*R*[*F* ^2^ > 2σ(*F* ^2^)] = 0.031
*wR*(*F* ^2^) = 0.072
*S* = 0.95
3851 reflections
325 parameters
H-atom parameters constrained
Δρ_max_ = 0.30 e Å^−3^
Δρ_min_ = −0.43 e Å^−3^

### 

Data collection: *APEX2* (Bruker, 2004 ▸); cell refinement: *SAINT* (Bruker, 2004 ▸); data reduction: *SAINT*; program(s) used to solve structure: *SHELXS97* (Sheldrick, 2008 ▸); program(s) used to refine structure: *SHELXL97* (Sheldrick, 2008 ▸); molecular graphics: *DIAMOND* (Brandenburg & Putz, 1999 ▸); software used to prepare material for publication: *SHELXTL* (Sheldrick, 2008 ▸).

## Supplementary Material

Crystal structure: contains datablock(s) I, New_Global_Publ_Block. DOI: 10.1107/S205698901500420X/zp2016sup1.cif

**Fig. 1.**
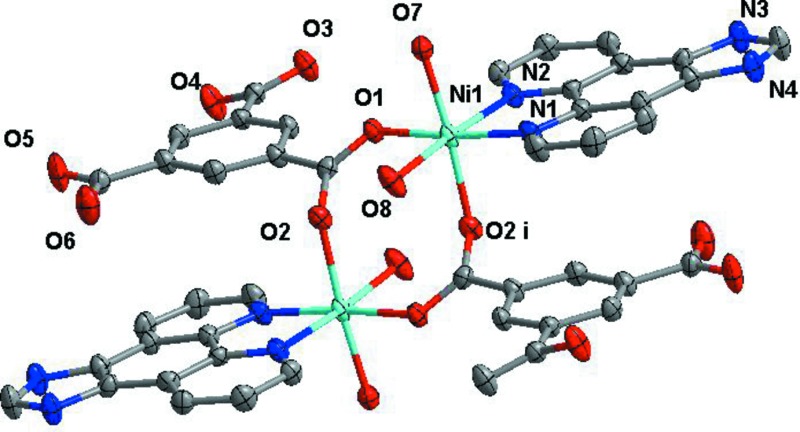
The molecular structure of the title compound, showing 30% probability displacement ellipsoids with the atom numbering. H atoms have been omitted for clarity. [Symmetry code: (i) -x + 1, -y + 1, -z + 1.]

**Fig. 2.**
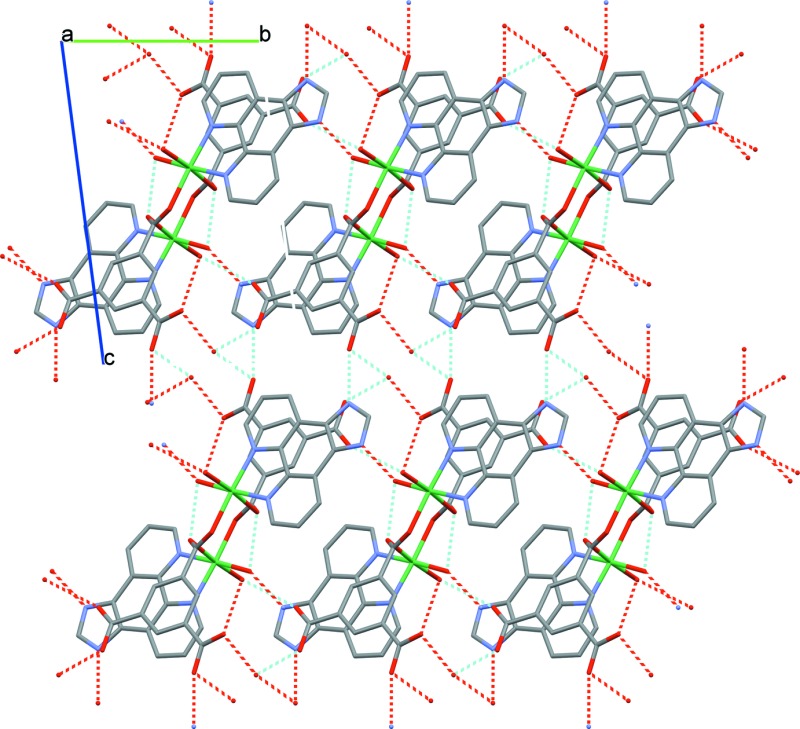
A packing view of the three-dimensional supermolecular framework of the title compound viewed along the a axis.

Structure factors: contains datablock(s) I. DOI: 10.1107/S205698901500420X/zp2016Isup2.hkl

x y z . DOI: 10.1107/S205698901500420X/zp2016fig1.tif
The mol­ecular structure of the title compound, showing 30% probability displacement ellipsoids with the atom numbering. H atoms have been omitted for clarity. [Symmetry code: (i) −*x* + 1, −*y* + 1, −*z* + 1.]

a . DOI: 10.1107/S205698901500420X/zp2016fig2.tif
A packing view of the three-dimensional supermolecular framework of the title compound viewed along the *a* axis.

CCDC reference: 1051597

Additional supporting information:  crystallographic information; 3D view; checkCIF report

## Figures and Tables

**Table 1:** Hydrogen-bond geometry (, )

*D*H*A*	*D*H	H*A*	*D* *A*	*D*H*A*
N4H4O6^i^	0.86	1.93	2.772(3)	165
O1H1*WA*O5^ii^	0.88	1.82	2.676(2)	165
O1H1*WB*O8^iii^	0.84	1.94	2.741(2)	161
O2H2*WA*N3^iv^	0.89	1.94	2.798(3)	160
O2H2*WB*O4	0.89	1.86	2.630(2)	144
O7H7*O*O9^iii^	0.85	1.72	2.558(2)	166
O9H9*WA*O5^ii^	0.86	1.88	2.684(2)	153
O9H9*WB*O6^v^	0.87	1.99	2.813(3)	159

Symmetry codes: (i) 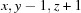; (ii) 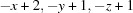; (iii) 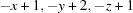; (iv) 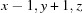; (v) 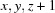.

## supplementary crystallographic information

### S1. Introduction

Imidazo[4,5-*f*][1,10]phenanthroline (IP) derivatives have been used to recognize the secondary structure of DNA in Ru(II) complexes. IP also an important heteroaromatic N-donor ligands for the construction of coordination polymers. A handful of comcpounds based on IP and carboxyl­ate ligands have been described (Liu *et al.*, 2009; Stephenson *et al.*, 2008; Wu *et al.*, 1997; Yang *et al.*, 2010). The title compound was prepared during an attempt to prepare a coordination polymer containing both benzene­tri­carboxyl­ate (BTC) and IP ligands, however, an simple dinuclear complex obtained.

### S2. Synthesis and crystallization

Nickel nitrate hexahydrate and benzene­tri­carboxyl­ate acid were obtained commercially. imidazo[4,5-*f*][1,10]phenanthroline was prepared *via* a published procedure (Wu *et al.* (1997). A mixture of Nickel nitrate hexahydrate (133 mg, 0.50 mmol), benzene­tri­carboxyl­ate acid (105 mg, 0.50 mmol), imidazo[4,5-*f*][1,10]phenanthroline (0.110 g,0.5 mmol) and 10.0 g water (550 mmol) was placed into a 23 ml Teflon-lined Parr Acid Digestion bomb, which was then heated under autogenous pressure at 398 K for 72 h, then cooled to RT at a rate of 5 °c/h. The resulting green crystals of the title compound were obtained.

### S3. Refinement

All H atoms were found in a difference Fourier map. The H atoms bound to C or N atoms were placed in calculated positions, with C—H= 0.93Å (CH) or N—H=0.86Å (NH)), *U*_iso_(H)= 1.2 times *U*_eq_(C or N). The H atoms bound to O atoms were restrained with O—H = 0.85 Å, and refined with *U*_iso_(H)= 1.5 times *U*_eq_(O).

### Crystal data

**Table:**

[Ni_2_(C_9_H_4_O_6_)_2_(C_13_H_8_N_4_)_2_(H_2_O)_4_]·2H_2_O	*Z* = 1
*M**_r_* = 1082.22	*F*(000) = 556
Triclinic, *P*1	*D*_x_ = 1.713 Mg m^−^^3^
Hall symbol: -P 1	Mo *K*α radiation, λ = 0.71069 Å
*a* = 8.581 (5) Å	Cell parameters from 2586 reflections
*b* = 9.032 (5) Å	θ = 3.6–24.9°
*c* = 14.278 (5) Å	µ = 0.99 mm^−^^1^
α = 82.222 (5)°	*T* = 293 K
β = 87.729 (5)°	Block, yellow-green
γ = 73.117 (5)°	0.28 × 0.16 × 0.15 mm
*V* = 1049.2 (9) Å^3^	

### Data collection

**Table:**

Bruker APEXII CCD diffractometer	3851 independent reflections
Radiation source: fine-focus sealed tube	3050 reflections with *I* > 2σ(*I*)
Graphite monochromator	*R*_int_ = 0.048
phi and ω scans	θ_max_ = 25.5°, θ_min_ = 2.4°
Absorption correction: multi-scan (*SADABS*; Bruker, 2004)	*h* = −6→10
*T*_min_ = 0.805, *T*_max_ = 0.867	*k* = −10→10
5594 measured reflections	*l* = −16→17

### Refinement

**Table:**

Refinement on *F*^2^	Primary atom site location: structure-invariant direct methods
Least-squares matrix: full	Secondary atom site location: difference Fourier map
*R*[*F*^2^ > 2σ(*F*^2^)] = 0.031	Hydrogen site location: inferred from neighbouring sites
*wR*(*F*^2^) = 0.072	H-atom parameters constrained
*S* = 0.95	*w* = 1/[σ^2^(*F*_o_^2^) + (0.0333*P*)^2^] where *P* = (*F*_o_^2^ + 2*F*_c_^2^)/3
3851 reflections	(Δ/σ)_max_ = 0.001
325 parameters	Δρ_max_ = 0.30 e Å^−^^3^
0 restraints	Δρ_min_ = −0.42 e Å^−^^3^

### Special details

**Table:**

Geometry. All s.u.'s (except the s.u. in the dihedral angle between two l.s. planes) are estimated using the full covariance matrix. The cell s.u.'s are taken into account individually in the estimation of s.u.'s in distances, angles and torsion angles; correlations between s.u.'s in cell parameters are only used when they are defined by crystal symmetry. An approximate (isotropic) treatment of cell s.u.'s is used for estimating s.u.'s involving l.s. planes.
Refinement. Refinement of *F*^2^ against ALL reflections. The weighted *R*-factor *wR* and goodness of fit *S* are based on *F*^2^, conventional *R*-factors *R* are based on *F*, with *F* set to zero for negative *F*^2^. The threshold expression of *F*^2^ > 2σ(*F*^2^) is used only for calculating *R*-factors(gt) *etc*. and is not relevant to the choice of reflections for refinement. *R*-factors based on *F*^2^ are statistically about twice as large as those based on *F*, and *R*- factors based on ALL data will be even larger.

### Fractional atomic coordinates and isotropic or equivalent isotropic displacement parameters (Å^2^)

**Table:**

	*x*	*y*	*z*	*U*_iso_*/*U*_eq_	
C1	0.6312 (3)	0.3200 (3)	0.81421 (15)	0.0288 (6)	
H1	0.5473	0.4127	0.8124	0.035*	
C2	0.6567 (3)	0.2172 (3)	0.89778 (16)	0.0338 (6)	
H2	0.5912	0.2413	0.9503	0.041*	
C3	0.7796 (3)	0.0801 (3)	0.90156 (16)	0.0310 (6)	
H3	0.7976	0.0092	0.9565	0.037*	
C4	0.8778 (3)	0.0478 (3)	0.82195 (15)	0.0227 (5)	
C5	0.8443 (3)	0.1596 (2)	0.74049 (15)	0.0209 (5)	
C6	0.9472 (3)	0.1389 (2)	0.65729 (15)	0.0207 (5)	
C7	1.0812 (3)	0.0055 (3)	0.65371 (15)	0.0231 (5)	
C8	1.1781 (3)	−0.0002 (3)	0.57196 (16)	0.0270 (5)	
H8	1.2676	−0.0861	0.5664	0.032*	
C9	1.1399 (3)	0.1215 (3)	0.50060 (16)	0.0280 (6)	
H9	1.2038	0.1190	0.4464	0.034*	
C10	1.0050 (3)	0.2493 (3)	0.50942 (15)	0.0258 (5)	
H10	0.9811	0.3315	0.4605	0.031*	
C11	1.1089 (3)	−0.1101 (3)	0.73572 (15)	0.0252 (5)	
C12	1.0117 (3)	−0.0873 (3)	0.81472 (15)	0.0243 (5)	
C13	1.2023 (3)	−0.3077 (3)	0.83840 (17)	0.0369 (6)	
H13	1.2656	−0.4031	0.8682	0.044*	
C14	0.5942 (3)	0.6540 (2)	0.43305 (15)	0.0220 (5)	
C15	0.6305 (3)	0.7419 (3)	0.34261 (15)	0.0212 (5)	
C16	0.7606 (3)	0.6785 (3)	0.28582 (15)	0.0234 (5)	
H16	0.8326	0.5814	0.3061	0.028*	
C17	0.7849 (3)	0.7588 (3)	0.19821 (15)	0.0223 (5)	
C18	0.6787 (3)	0.9059 (3)	0.17046 (16)	0.0264 (5)	
H18	0.6933	0.9601	0.1122	0.032*	
C19	0.5521 (3)	0.9727 (3)	0.22814 (15)	0.0243 (5)	
C20	0.5259 (3)	0.8897 (3)	0.31353 (15)	0.0249 (5)	
H20	0.4384	0.9329	0.3515	0.030*	
C22	0.9187 (3)	0.6874 (3)	0.13265 (15)	0.0240 (5)	
C23	0.4356 (3)	1.1296 (3)	0.19953 (17)	0.0288 (6)	
Ni1	0.70279 (4)	0.43502 (3)	0.609962 (19)	0.02230 (10)	
N1	0.7200 (2)	0.2927 (2)	0.73743 (12)	0.0229 (4)	
N2	0.9089 (2)	0.2582 (2)	0.58513 (12)	0.0217 (4)	
N3	1.2298 (2)	−0.2516 (2)	0.75156 (13)	0.0329 (5)	
N4	1.0757 (2)	−0.2163 (2)	0.88009 (13)	0.0315 (5)	
H4	1.0409	−0.2341	0.9366	0.038*	
O1	0.83799 (18)	0.55829 (17)	0.66524 (10)	0.0274 (4)	
H1WA	0.8974	0.5175	0.7162	0.041*	
H1WB	0.7704	0.6288	0.6903	0.041*	
O2	0.48839 (19)	0.60135 (19)	0.63947 (11)	0.0368 (4)	
H2WA	0.4226	0.6611	0.6785	0.055*	
H2WB	0.4306	0.6295	0.5865	0.055*	
O3	0.71191 (18)	0.56435 (17)	0.48112 (10)	0.0257 (4)	
O4	0.44422 (18)	0.67849 (17)	0.45611 (10)	0.0249 (4)	
O5	1.0320 (2)	0.57271 (19)	0.16548 (11)	0.0346 (4)	
O6	0.9071 (2)	0.74669 (19)	0.04712 (11)	0.0365 (4)	
O7	0.4671 (2)	1.19600 (19)	0.11669 (12)	0.0474 (5)	
H7O	0.3828	1.2679	0.0956	0.071*	
O8	0.3214 (2)	1.18626 (19)	0.24846 (12)	0.0436 (5)	
O9	0.7523 (2)	0.56589 (19)	0.96006 (11)	0.0404 (5)	
H9WA	0.8263	0.5505	0.9168	0.061*	
H9WB	0.7762	0.6295	0.9933	0.061*	

### Atomic displacement parameters (Å^2^)

**Table:**

	*U*^11^	*U*^22^	*U*^33^	*U*^12^	*U*^13^	*U*^23^
C1	0.0238 (13)	0.0318 (14)	0.0252 (13)	−0.0005 (11)	0.0062 (11)	−0.0021 (10)
C2	0.0336 (15)	0.0396 (15)	0.0225 (12)	−0.0044 (12)	0.0105 (11)	−0.0014 (11)
C3	0.0342 (15)	0.0327 (14)	0.0205 (12)	−0.0051 (12)	0.0044 (11)	0.0044 (10)
C4	0.0225 (12)	0.0254 (12)	0.0193 (11)	−0.0073 (10)	0.0023 (10)	0.0007 (9)
C5	0.0202 (12)	0.0238 (12)	0.0181 (11)	−0.0062 (10)	0.0009 (10)	−0.0007 (9)
C6	0.0190 (12)	0.0242 (12)	0.0186 (11)	−0.0064 (10)	0.0004 (9)	−0.0010 (9)
C7	0.0211 (12)	0.0260 (12)	0.0216 (12)	−0.0066 (10)	0.0024 (10)	−0.0027 (10)
C8	0.0240 (13)	0.0257 (13)	0.0286 (13)	−0.0039 (11)	0.0050 (11)	−0.0028 (10)
C9	0.0266 (13)	0.0334 (14)	0.0217 (12)	−0.0068 (11)	0.0088 (10)	−0.0021 (10)
C10	0.0276 (13)	0.0297 (13)	0.0192 (11)	−0.0090 (11)	0.0029 (10)	0.0012 (10)
C11	0.0252 (13)	0.0224 (12)	0.0252 (12)	−0.0040 (11)	0.0023 (11)	−0.0001 (10)
C12	0.0249 (13)	0.0258 (12)	0.0201 (11)	−0.0061 (11)	0.0023 (10)	0.0009 (10)
C13	0.0355 (15)	0.0277 (14)	0.0354 (15)	0.0038 (12)	0.0040 (12)	0.0084 (11)
C14	0.0252 (13)	0.0221 (12)	0.0179 (11)	−0.0054 (11)	0.0030 (10)	−0.0034 (9)
C15	0.0205 (12)	0.0250 (12)	0.0171 (11)	−0.0063 (10)	0.0003 (10)	0.0005 (9)
C16	0.0212 (12)	0.0223 (12)	0.0224 (12)	−0.0016 (10)	−0.0021 (10)	0.0024 (9)
C17	0.0197 (12)	0.0258 (12)	0.0198 (11)	−0.0051 (10)	0.0002 (10)	0.0003 (9)
C18	0.0273 (13)	0.0277 (13)	0.0208 (12)	−0.0062 (11)	0.0017 (10)	0.0037 (10)
C19	0.0229 (13)	0.0240 (12)	0.0235 (12)	−0.0046 (11)	−0.0003 (10)	0.0013 (10)
C20	0.0214 (12)	0.0286 (13)	0.0228 (12)	−0.0045 (11)	0.0051 (10)	−0.0041 (10)
C22	0.0237 (13)	0.0253 (13)	0.0224 (12)	−0.0071 (11)	0.0016 (10)	−0.0010 (10)
C23	0.0291 (14)	0.0255 (13)	0.0290 (13)	−0.0050 (11)	−0.0001 (12)	−0.0003 (11)
Ni1	0.01924 (16)	0.02540 (17)	0.01782 (16)	−0.00195 (13)	0.00227 (12)	0.00200 (12)
N1	0.0193 (10)	0.0253 (10)	0.0215 (10)	−0.0036 (9)	0.0017 (8)	−0.0006 (8)
N2	0.0219 (10)	0.0243 (10)	0.0166 (9)	−0.0055 (9)	0.0021 (8)	0.0017 (8)
N3	0.0295 (12)	0.0291 (11)	0.0283 (11)	0.0053 (10)	0.0070 (9)	0.0043 (9)
N4	0.0333 (12)	0.0313 (12)	0.0214 (10)	−0.0019 (10)	0.0070 (9)	0.0077 (9)
O1	0.0260 (9)	0.0286 (9)	0.0230 (8)	−0.0014 (7)	−0.0001 (7)	−0.0022 (7)
O2	0.0285 (10)	0.0466 (11)	0.0211 (9)	0.0106 (8)	0.0009 (7)	−0.0027 (8)
O3	0.0202 (8)	0.0312 (9)	0.0202 (8)	−0.0030 (7)	0.0009 (7)	0.0065 (7)
O4	0.0200 (8)	0.0314 (9)	0.0201 (8)	−0.0044 (7)	0.0038 (7)	−0.0001 (7)
O5	0.0287 (10)	0.0391 (10)	0.0238 (9)	0.0074 (8)	0.0002 (7)	0.0006 (8)
O6	0.0357 (10)	0.0424 (11)	0.0201 (9)	0.0012 (9)	0.0069 (8)	0.0063 (8)
O7	0.0398 (11)	0.0397 (11)	0.0407 (11)	0.0114 (9)	0.0098 (9)	0.0172 (9)
O8	0.0469 (12)	0.0290 (10)	0.0425 (11)	0.0061 (9)	0.0138 (10)	−0.0032 (8)
O9	0.0411 (11)	0.0382 (10)	0.0320 (10)	0.0021 (9)	0.0049 (8)	−0.0016 (8)

### Geometric parameters (Å, º)

**Table:**

C1—N1	1.319 (3)	C15—C16	1.384 (3)
C1—C2	1.392 (3)	C15—C20	1.394 (3)
C1—H1	0.9300	C16—C17	1.399 (3)
C2—C3	1.370 (3)	C16—H16	0.9300
C2—H2	0.9300	C17—C18	1.391 (3)
C3—C4	1.399 (3)	C17—C22	1.503 (3)
C3—H3	0.9300	C18—C19	1.381 (3)
C4—C5	1.413 (3)	C18—H18	0.9300
C4—C12	1.425 (3)	C19—C20	1.388 (3)
C5—N1	1.353 (3)	C19—C23	1.492 (3)
C5—C6	1.451 (3)	C20—H20	0.9300
C6—N2	1.361 (3)	C22—O5	1.248 (3)
C6—C7	1.408 (3)	C22—O6	1.261 (3)
C7—C8	1.404 (3)	C23—O8	1.209 (3)
C7—C11	1.437 (3)	C23—O7	1.307 (3)
C8—C9	1.367 (3)	Ni1—O3	2.0531 (16)
C8—H8	0.9300	Ni1—N1	2.0652 (19)
C9—C10	1.392 (3)	Ni1—N2	2.066 (2)
C9—H9	0.9300	Ni1—O1	2.0662 (17)
C10—N2	1.330 (3)	Ni1—O2	2.0794 (18)
C10—H10	0.9300	Ni1—O4^i^	2.1540 (17)
C11—C12	1.378 (3)	N4—H4	0.8600
C11—N3	1.390 (3)	O1—H1WA	0.8794
C12—N4	1.380 (3)	O1—H1WB	0.8376
C13—N3	1.314 (3)	O2—H2WA	0.8944
C13—N4	1.333 (3)	O2—H2WB	0.8873
C13—H13	0.9300	O4—Ni1^i^	2.1540 (17)
C14—O3	1.255 (3)	O7—H7O	0.8528
C14—O4	1.279 (3)	O9—H9WA	0.8627
C14—C15	1.493 (3)	O9—H9WB	0.8651
			
N1—C1—C2	123.3 (2)	C19—C18—C17	121.0 (2)
N1—C1—H1	118.3	C19—C18—H18	119.5
C2—C1—H1	118.3	C17—C18—H18	119.5
C3—C2—C1	119.0 (2)	C18—C19—C20	119.7 (2)
C3—C2—H2	120.5	C18—C19—C23	122.0 (2)
C1—C2—H2	120.5	C20—C19—C23	118.2 (2)
C2—C3—C4	119.3 (2)	C19—C20—C15	120.2 (2)
C2—C3—H3	120.3	C19—C20—H20	119.9
C4—C3—H3	120.3	C15—C20—H20	119.9
C3—C4—C5	117.8 (2)	O5—C22—O6	124.3 (2)
C3—C4—C12	126.4 (2)	O5—C22—C17	118.4 (2)
C5—C4—C12	115.84 (19)	O6—C22—C17	117.2 (2)
N1—C5—C4	122.0 (2)	O8—C23—O7	124.1 (2)
N1—C5—C6	117.03 (19)	O8—C23—C19	122.2 (2)
C4—C5—C6	120.9 (2)	O7—C23—C19	113.7 (2)
N2—C6—C7	122.7 (2)	O3—Ni1—N1	173.78 (7)
N2—C6—C5	115.78 (19)	O3—Ni1—N2	94.05 (7)
C7—C6—C5	121.52 (19)	N1—Ni1—N2	80.10 (7)
C8—C7—C6	117.2 (2)	O3—Ni1—O1	88.33 (7)
C8—C7—C11	126.1 (2)	N1—Ni1—O1	89.79 (7)
C6—C7—C11	116.7 (2)	N2—Ni1—O1	92.10 (8)
C9—C8—C7	119.5 (2)	O3—Ni1—O2	89.01 (6)
C9—C8—H8	120.2	N1—Ni1—O2	96.93 (7)
C7—C8—H8	120.2	N2—Ni1—O2	176.09 (7)
C8—C9—C10	119.8 (2)	O1—Ni1—O2	90.43 (8)
C8—C9—H9	120.1	O3—Ni1—O4^i^	87.63 (6)
C10—C9—H9	120.1	N1—Ni1—O4^i^	94.41 (7)
N2—C10—C9	122.6 (2)	N2—Ni1—O4^i^	89.96 (8)
N2—C10—H10	118.7	O1—Ni1—O4^i^	175.58 (6)
C9—C10—H10	118.7	O2—Ni1—O4^i^	87.72 (8)
C12—C11—N3	109.89 (19)	C1—N1—C5	118.49 (19)
C12—C11—C7	121.0 (2)	C1—N1—Ni1	128.11 (16)
N3—C11—C7	129.1 (2)	C5—N1—Ni1	113.32 (14)
C11—C12—N4	105.6 (2)	C10—N2—C6	118.2 (2)
C11—C12—C4	123.9 (2)	C10—N2—Ni1	128.14 (16)
N4—C12—C4	130.4 (2)	C6—N2—Ni1	113.64 (14)
N3—C13—N4	114.2 (2)	C13—N3—C11	103.84 (19)
N3—C13—H13	122.9	C13—N4—C12	106.45 (19)
N4—C13—H13	122.9	C13—N4—H4	126.8
O3—C14—O4	125.1 (2)	C12—N4—H4	126.8
O3—C14—C15	118.1 (2)	Ni1—O1—H1WA	120.3
O4—C14—C15	116.79 (19)	Ni1—O1—H1WB	105.6
C16—C15—C20	119.6 (2)	H1WA—O1—H1WB	96.1
C16—C15—C14	121.87 (19)	Ni1—O2—H2WA	152.1
C20—C15—C14	118.51 (19)	Ni1—O2—H2WB	106.7
C15—C16—C17	120.7 (2)	H2WA—O2—H2WB	101.2
C15—C16—H16	119.7	C14—O3—Ni1	127.43 (14)
C17—C16—H16	119.7	C14—O4—Ni1^i^	119.27 (14)
C18—C17—C16	118.7 (2)	C23—O7—H7O	109.8
C18—C17—C22	119.7 (2)	H9WA—O9—H9WB	105.2
C16—C17—C22	121.5 (2)		
			
N1—C1—C2—C3	−0.1 (4)	C18—C19—C23—O8	177.5 (2)
C1—C2—C3—C4	0.9 (4)	C20—C19—C23—O8	0.7 (4)
C2—C3—C4—C5	−0.2 (4)	C18—C19—C23—O7	−0.9 (3)
C2—C3—C4—C12	178.8 (2)	C20—C19—C23—O7	−177.7 (2)
C3—C4—C5—N1	−1.4 (3)	C2—C1—N1—C5	−1.4 (4)
C12—C4—C5—N1	179.49 (19)	C2—C1—N1—Ni1	−177.93 (17)
C3—C4—C5—C6	176.1 (2)	C4—C5—N1—C1	2.2 (3)
C12—C4—C5—C6	−3.1 (3)	C6—C5—N1—C1	−175.4 (2)
N1—C5—C6—N2	1.3 (3)	C4—C5—N1—Ni1	179.18 (17)
C4—C5—C6—N2	−176.32 (19)	C6—C5—N1—Ni1	1.6 (2)
N1—C5—C6—C7	179.14 (19)	O3—Ni1—N1—C1	154.2 (5)
C4—C5—C6—C7	1.6 (3)	N2—Ni1—N1—C1	174.0 (2)
N2—C6—C7—C8	0.9 (3)	O1—Ni1—N1—C1	81.8 (2)
C5—C6—C7—C8	−176.9 (2)	O2—Ni1—N1—C1	−8.6 (2)
N2—C6—C7—C11	178.9 (2)	O4^i^—Ni1—N1—C1	−96.8 (2)
C5—C6—C7—C11	1.1 (3)	O3—Ni1—N1—C5	−22.5 (7)
C6—C7—C8—C9	0.2 (3)	N2—Ni1—N1—C5	−2.66 (15)
C11—C7—C8—C9	−177.6 (2)	O1—Ni1—N1—C5	−94.83 (16)
C7—C8—C9—C10	−0.4 (4)	O2—Ni1—N1—C5	174.76 (15)
C8—C9—C10—N2	−0.5 (4)	O4^i^—Ni1—N1—C5	86.53 (15)
C8—C7—C11—C12	175.6 (2)	C9—C10—N2—C6	1.5 (3)
C6—C7—C11—C12	−2.2 (3)	C9—C10—N2—Ni1	−178.89 (16)
C8—C7—C11—N3	−2.5 (4)	C7—C6—N2—C10	−1.7 (3)
C6—C7—C11—N3	179.7 (2)	C5—C6—N2—C10	176.17 (19)
N3—C11—C12—N4	0.7 (3)	C7—C6—N2—Ni1	178.65 (16)
C7—C11—C12—N4	−177.7 (2)	C5—C6—N2—Ni1	−3.5 (2)
N3—C11—C12—C4	179.1 (2)	O3—Ni1—N2—C10	1.62 (19)
C7—C11—C12—C4	0.7 (4)	N1—Ni1—N2—C10	−176.3 (2)
C3—C4—C12—C11	−177.0 (2)	O1—Ni1—N2—C10	−86.85 (19)
C5—C4—C12—C11	2.0 (3)	O2—Ni1—N2—C10	142.9 (9)
C3—C4—C12—N4	0.9 (4)	O4^i^—Ni1—N2—C10	89.23 (19)
C5—C4—C12—N4	179.9 (2)	O3—Ni1—N2—C6	−178.76 (15)
O3—C14—C15—C16	34.7 (3)	N1—Ni1—N2—C6	3.35 (15)
O4—C14—C15—C16	−146.4 (2)	O1—Ni1—N2—C6	92.77 (15)
O3—C14—C15—C20	−147.8 (2)	O2—Ni1—N2—C6	−37.4 (10)
O4—C14—C15—C20	31.1 (3)	O4^i^—Ni1—N2—C6	−91.14 (15)
C20—C15—C16—C17	−2.2 (3)	N4—C13—N3—C11	−0.1 (3)
C14—C15—C16—C17	175.4 (2)	C12—C11—N3—C13	−0.4 (3)
C15—C16—C17—C18	2.0 (3)	C7—C11—N3—C13	177.8 (2)
C15—C16—C17—C22	−175.8 (2)	N3—C13—N4—C12	0.5 (3)
C16—C17—C18—C19	0.4 (3)	C11—C12—N4—C13	−0.7 (3)
C22—C17—C18—C19	178.3 (2)	C4—C12—N4—C13	−178.9 (2)
C17—C18—C19—C20	−2.6 (3)	O4—C14—O3—Ni1	−1.4 (3)
C17—C18—C19—C23	−179.4 (2)	C15—C14—O3—Ni1	177.45 (13)
C18—C19—C20—C15	2.3 (3)	N1—Ni1—O3—C14	168.8 (6)
C23—C19—C20—C15	179.2 (2)	N2—Ni1—O3—C14	149.23 (18)
C16—C15—C20—C19	0.0 (3)	O1—Ni1—O3—C14	−118.78 (18)
C14—C15—C20—C19	−177.6 (2)	O2—Ni1—O3—C14	−28.32 (18)
C18—C17—C22—O5	165.0 (2)	O4^i^—Ni1—O3—C14	59.44 (18)
C16—C17—C22—O5	−17.2 (3)	O3—C14—O4—Ni1^i^	−101.9 (2)
C18—C17—C22—O6	−16.0 (3)	C15—C14—O4—Ni1^i^	79.2 (2)
C16—C17—C22—O6	161.7 (2)		

Symmetry code: (i) −*x*+1, −*y*+1, −*z*+1.

### Hydrogen-bond geometry (Å, º)

**Table:**

*D*—H···*A*	*D*—H	H···*A*	*D*···*A*	*D*—H···*A*
N4—H4···O6^ii^	0.86	1.93	2.772 (3)	165
O1—H1*WA*···O5^iii^	0.88	1.82	2.676 (2)	165
O1—H1*WB*···O8^iv^	0.84	1.94	2.741 (2)	161
O2—H2*WA*···N3^v^	0.89	1.94	2.798 (3)	160
O2—H2*WB*···O4	0.89	1.86	2.630 (2)	144
O7—H7*O*···O9^iv^	0.85	1.72	2.558 (2)	166
O9—H9*WA*···O5^iii^	0.86	1.88	2.684 (2)	153
O9—H9*WB*···O6^vi^	0.87	1.99	2.813 (3)	159

Symmetry codes: (ii) *x*, *y*−1, *z*+1; (iii) −*x*+2, −*y*+1, −*z*+1; (iv) −*x*+1, −*y*+2, −*z*+1; (v) *x*−1, *y*+1, *z*; (vi) *x*, *y*, *z*+1.
