# Supplementary figures and images for: Crystal structure of a binuclear nickel(II) complex constructed of 1H-imidazo[4,5-f][1,10]phenanthroline and doubly deprotonated benzene-1,3,5-tri­carb­oxy­lic acid
Source: Acta Crystallogr E Crystallogr Commun. 2015 Mar 21;71(Pt 4):m95–6. doi: 10.1107/S205698901500420X (PMC4438790; doi:10.1107/S205698901500420X)

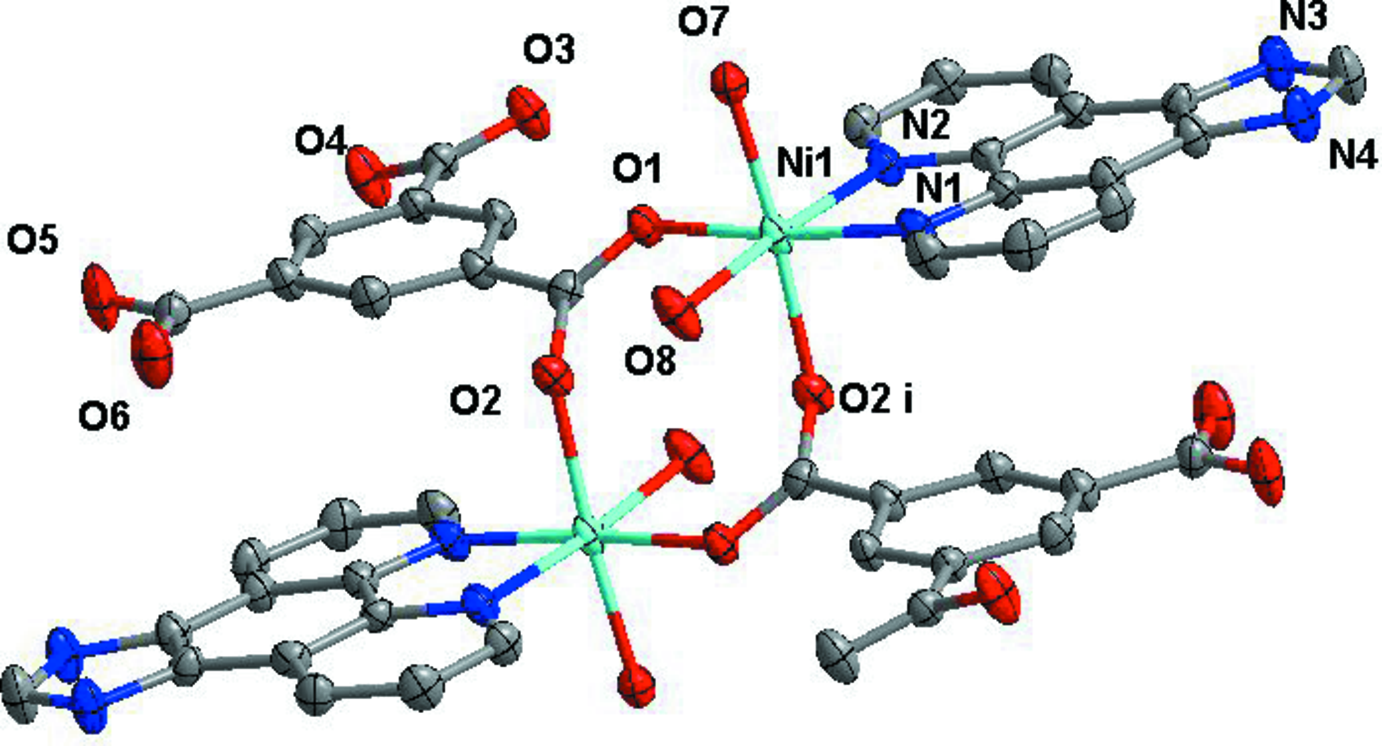

Supplement: Supplementary file 3 [file e-71-00m95-fig1.tif]

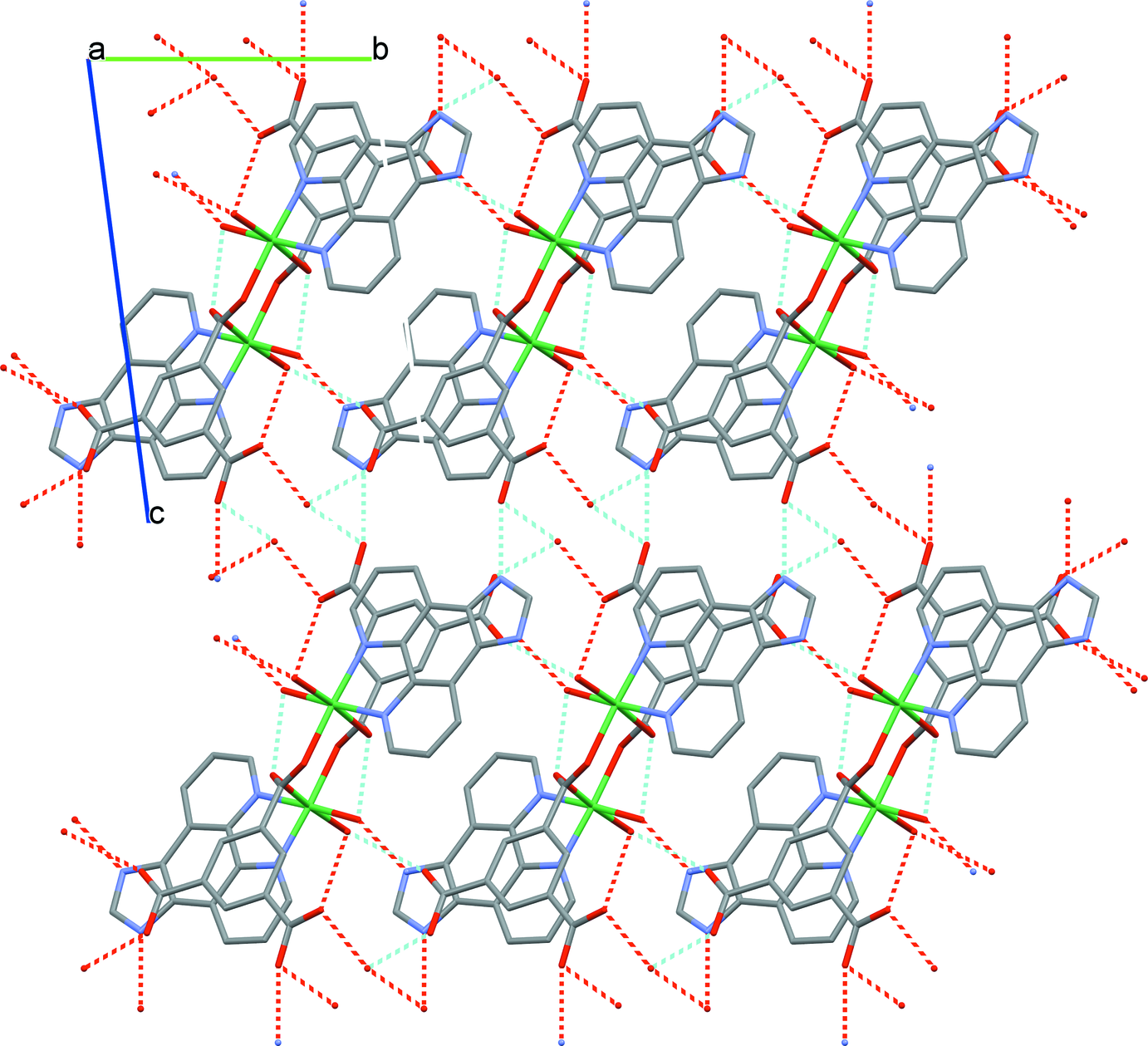

Supplement: Supplementary file 4 [file e-71-00m95-fig2.tif]
